# Leukocyte and IgM Responses to Immunization with the CIDR1α-PfEMP1 Recombinant Protein in the Wistar Rat

**DOI:** 10.3390/tropicalmed7090222

**Published:** 2022-09-02

**Authors:** Erma Sulistyaningsih, Renaldi Wibisono, Rosita Dewi

**Affiliations:** 1 Department of Parasitology, Faculty of Medicine, University of Jember, Jember 68121, Indonesia; 2 Center of Excellence of Agromedicine (CEAMED), University of Jember, Jember 68121, Indonesia; 3 Medical Science Program, Faculty of Medicine, University of Jember, Jember 68121, Indonesia; 4 Department of Histology, Faculty of Medicine, University of Jember, Jember 68121, Indonesia

**Keywords:** CIDR1α, IgM, leukocyte, malaria, PfEMP1, *Plasmodium falciparum*, vaccine

## Abstract

The malaria vaccine is an important strategy for the global malaria elimination program, but the complexity of the *Plasmodium* antigen is a major hurdle in malaria vaccine development. The cysteine-rich interdomain region 1α (CIDR1α) of *Plasmodium falciparum* erythrocyte membrane protein 1 (PfEMP1) is crucial in malaria pathogenesis, making it a vaccine candidate. This study investigated the leukocyte and IgM response generated after administering a CIDR1α-PfEMP1 recombinant protein injection in Wistar rats. The rats were divided into a control group, who received a physiological saline solution (PSS), and a treatment group, who were subcutaneously injected with 150 µg of purified CIDR1α-PfEMP1 protein three times at the 3-week interval. Blood samples were collected every week after each injection. The number of leukocytes were counted using a Neubauer chamber, and the IgM concentration was determined using an enzyme-linked immunosorbent assay (ELISA). Data were analyzed using an independent, paired-T test, a Mann–Whitney test, and a Wilcoxon test, based on the distribution of the data. The total number of leukocytes notably increased on day 29 (*p* < 0.05). The percentage of neutrophils decreased, especially on day 8 (*p* < 0.05), whereas the percentages of monocytes and lymphocytes increased, primarily on day 14 (*p* < 0.05). The IgM concentration increased on day 14 (*p* < 0.05). In conclusion, the CIDR1α-PfEMP1 recombinant protein may induce leukocyte and IgM responses, making it a potential malaria vaccine candidate.

## 1. Introduction

Malaria is an infectious disease caused by *Plasmodium spp. falciparum* and is transmitted by female *Anopheles* mosquitoes [1]. There are five *Plasmodium* spp. causing human malaria, but the majority are caused by the most deadly species: *P. falciparum.* This pathogen accounts for 90% of malaria deaths in Africa, mostly among children [2]. In 2020, the World Health Organization (WHO) reported that there were an estimated 241 million cases of malaria in 85 tropical and subtropical countries. The incidence and mortality of malaria cases reached 59 and 15 cases (per 1000 populations at risk), respectively [3]. Indonesia accounted for 10.4% of malaria cases in the South East Asia Region, with an estimated 226,364 cases in 2020 and an annual parasite incidence of 0.9% [4].

There are global and regional commitments to accelerating malaria elimination, however some challenges include an increased resistance to antimalarial drugs and insecticides. Therefore, the malaria vaccine could be a pivotal strategy for preventing and controlling the disease [5]. The major problem in designing an effective malaria vaccine is the complexity and variety of the *Plasmodium* antigens. The most successful malaria vaccine development attempt has been the RTS,S/AS01 vaccine, which was designed based on the circumsporozoite protein. In October 2021, WHO approved the use of this vaccine for children in malaria-endemic areas. However, this vaccine provides 34.8% protection against severe malaria [6]. Therefore, developing a malaria vaccine using another potential immunogenic protein is crucial. One of the malaria vaccine candidates currently being developed is the cysteine-rich interdomain region 1α (CIDR1α) of *Plasmodium falciparum* erythrocyte membrane protein 1 (PfEMP1), which plays an imperative role in the pathogenesis of malaria [7].

The CIDR1α domain is a part of the PfEMP1 protein that is secreted by *P. falciparum* during the erythrocytic cycle and is expressed on the infected erythrocyte (IE) surface [7]. The CIDR1α-PfEMP1 domain is involved in the sequestration and adhesion process by binding to host receptors, i.e., endothelial protein C receptor (EPCR) and cluster of differentiation 36 (CD36), leading to circulatory disturbance, vascular occlusion, inflammation, and death [8]. A previous study revealed that the CIDR1α domain mediates binding to CD36 on the surface of the leukocytes acting against the malaria infection [9]. Furthermore, an in silico study indicated that binding of the CIDR1α domain to the B-cell epitope induced the secretion of antibodies [7]. These studies bolster the potential of CIDR1α-PfEMP1 as a malaria vaccine candidate.

The immune response to malaria infection begins with the innate immunity and involves leukocytes. The leukocyte response contributes to the killing of pathogens by phagocytosis and by exposing components of the parasites [10,11,12]. Antigen-presenting cells (APCs) are capable of recognizing and projecting the exposed parasite components that are bound by major histocompatibility complex (MHC) [13]. Following activation of the APCs, the adaptive immune response is activated, involving T-cells, B-cells, and antibodies. Immunoglobulin M (IgM) mediates parasite clearance through complement-dependent opsonization, phagocytosis, and dendritic cell activation [14]. Therefore, leukocyte- and IgM-mediated immune responses could potentially be useful for determining antigens in the development of a malaria vaccine. Previous studies have constructed and expressed the CIDR1α-PfEMP1 recombinant protein from Indonesian isolates [15,16]. This study investigated leukocyte and IgM immune responses after immunization with the CIDR1α-PfEMP1 recombinant protein in Wistar rats.

## 2. Materials and Methods

### 2.1. Ethical Clearance

This study has been approved by the Research Ethics Committee in the Faculty of Medicine at the University of Jember, with a reference number of 1587/H25.1.11/KE/2022. This study used Wistar rats as the animal model.

### 2.2. Production and Purification of CIDR1α-PfEMP1 Recombinant Protein

The CIDR1α-PfEMP1 domain was amplified by polymerase chain reaction (PCR) using HotStar Taq DNA Polymerase with a specific primer consisting of CIDR1α_Fw 5′- CGGGATCCAAATGGAAATG TTATTATG -3′ for a BamHI site and CIDR1α_Rev 5′- CCCTCGAGTTGTAGTAATT TATCAATT -3′ for an XhoI site. The sequencing of the CIDR1α-PfEMP1 amplicon resulted in 525 nucleotides, which were subsequently fully expressed as 175 amino acids. The amplicon was ligated into the expression vector pET-30a. Furthermore, pET-30a-CIDR1α was transformed into *Escherichia coli* BL21 (DE3) competent cells using the heat shock method, as described by Dewi et al. [15].

*E. coli* BL21 (DE3) cells were cultured in Luria–Bertani (Liofilchem S.r.l., Teramo, Italy) and were combined with 50 μg/mL kanamycin (Thermo Fisher Scientific Inc., Waltham, MA, USA) at 37 °C by shaking at 190 rpm until the optical density of the sample was 600 nm (OD_600_) 0.6–0.8. The culture was induced with 0.3 mM isopropyl β-D-1-thiogalactopyranoside (IPTG) (Promega Co., Madison, WI, USA) by shaking at 190 rpm for 8 h at room temperature (RT) and was harvested through centrifugation for 15 min at 4 °C. The pellet was solubilized using an extraction buffer (300 mM NaCl, 50 mM NaH_2_PO_4_, and 5 mM imidazole in a pH of 8.0) and was incubated with 1 mg/mL lysozyme (VWR International LLC, Radnor, PA, USA) for 30 min at 4 °C [16].

As per the manufacturer’s protocol, the soluble fraction was purified by Ni-NTA agarose based on affinity chromatography. The soluble fraction was added to a column containing 1 mL of Ni-NTA resin (Qiagen, Hilden, Germany) and was washed with 1 mL of wash buffer I containing 300 mM NaCl, 50 mM NaH_2_PO_4_ and 20 mM imidazole in a pH of 8.0 twice and wash buffer II containing 300 mM NaCl, 50 mM NaH_2_PO_4,_ and 50 mM imidazole in a pH of 8.0 twice. The recombinant protein was eluted in a stepwise manner using 0.5 mL of an elution buffer (300 mM NaCl, 50 mM NaH_2_PO_4_ in a pH of 8.0) containing 80 mM, 100 mM, or 120 mM imidazole (Sigma-Aldrich Co., St. Louis, MO, USA) [17].

The purified proteins were visualized using SDS-PAGE (Bio-Rad Laboratories Inc., Hercules, CA, USA) at 80 V for 1.5 h. The gel was stained using Coomassie brilliant blue [18]. The concentration of CIDR1α-PfEMP1 recombinant protein was measured using the Bradford protein assay (HiMedia Laboratories, Maharashtra, India), as per the manufacturer’s protocol. The absorbance at 595 nm was measured using a spectrophotometer [16].

### 2.3. Immunization of CIDR1α-PfEMP1 Recombinant Protein in Wistar Rats

Twelve male Wistar rats, aged 2–3 months and weighing approximately 200–250 g, were used for the CIDR1α-PfEMP1 recombinant protein immunogenicity test. Rat cages were made from plastic with wire mesh on top. The floors of the cages were covered by wood shavings with a depth of 2 cm. The cages were rectangular, with a dimension of 40 × 30 × 13 cm (L × W × H). There was one cage for each rat. The rats were fed on commercial rat pellets and water ad libitum.

The rats were randomly distributed into two groups, i.e., a control and a treatment group. Each rat in the control group was subcutaneously injected with a physiological saline solution (PSS), and the rats in the treatment group were injected with 0.6–0.75 μg/g BW of purified CIDR1α-PfEMP1 protein. A total volume of 400 μL of this solution was prepared by dissolving 150 μg of purified CIDR1α-PfEMP1 protein in 200 μL phosphate-buffer saline (PBS) and emulsifying it in Freund’s adjuvant (Santa Cruz Biotechnology Inc., Dallas, TX, USA) using a 1:1 ratio. Complete Freund’s adjuvant was used for the primary immunization and incomplete Freund’s adjuvant was used for the subsequent immunizations. Rats were immunized three times in the 3-week interval (Day 0, 21, and 42) [19]. Blood samples were collected from the retro-orbital sinuses before immunization and every week after immunization (Day 0, 8, 14, 29, 35, 50, and 56). The rats were anesthetized with ketamine and xylazine via the intramuscular route and were topically anesthetized with proparacaine before retro-orbital sampling. On Day 56, the rats were anesthetized with ketamine and xylazine via the intramuscular route, and intracardiac blood samples were taken from the animals [20].

### 2.4. Measurement of Total Leukocyte and Leukocyte Differential Count

The blood samples were measured for the total number of leukocytes and the leukocyte differential count. Blood samples were diluted at a 1:20 ratio with Turk’s solution in a leukocyte Thoma pipette. The pipettes were rotated horizontally in order to homogenize the samples for 2–3 min. The improved Neubauer counting chamber was filled with the samples in order to count the total number of leukocytes using a Leica DM500 microscope at 40× magnification [21].

Blood smears were made on encoded clean slides, were fixed with methanol for 2–3 min, and were stained with *Giemsa* for 20 min. The leukocyte differential count was carried out using a Leica DM500 microscope at 40× magnification. The number of neutrophils, eosinophils, basophils, monocytes, and lymphocytes were counted in 100 cells using a differential blood cell counter [22].

### 2.5. Measurement of IgM Concentration

The IgM concentration of rat sera was determined using sandwich enzyme-linked immunosorbent assay (ELISA) methods, as per the manufacturer’s protocol (BT-Lab, Shanghai, China). This assay delivers 2–5 times higher sensitivity than other types of ELISA. The rat sera samples were collected two weeks after immunization (Day 14, 35, and 56). The ELISA plates were coated with rat IgM antibody and blocked with blocking solutions by the manufacturer. The procedure began by adding 40 μL of rat sera (no dilution required) into the wells. Following this, 10 μL of biotinylated rat IgM antibody and 50 μL of streptavidin-HRP were added. The plates were incubated at 37 °C for 60 min and were washed 5 times using wash buffer (50 mM Tris, 140 mM NaCl, 0.05% Tween 20 in a pH of 8.0) for 30 s. Substrate solutions were added to the wells and were incubated for 10 min at 37 °C in the dark. After adding 50 μL of stop solution (180 mM H_2_SO_4_), each sample was measured using an R-Biopharm ELISA reader at an absorbance of 450 nm within 10 min [23].

### 2.6. Statistical Analysis

The data were statistically analyzed using Statistical Package for the Social Sciences (SPSS) version 28.0 software (New York, NY, USA). The distribution of the dataset was analyzed using the Shapiro–Wilk test, and the homogeneity of the dataset was analyzed using Levene’s test. The data with normal distribution were analyzed using independent and paired *t*-tests, whereas the data with abnormal distribution were analyzed using Mann–Whitney and Wilcoxon tests with a confidence interval of 95%.

## 3. Results

### 3.1. Purification and Concentration of CIDR1α-PfEMP1 Recombinant Protein

**Figure 1:**
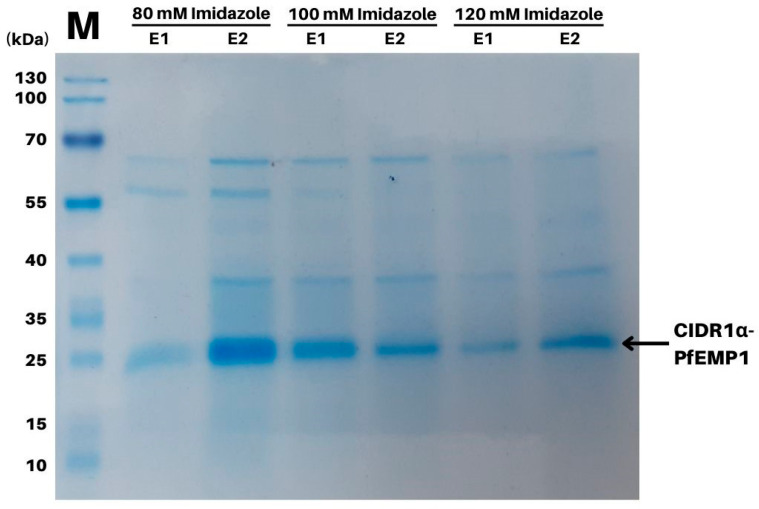
SDS-PAGE electrophoretogram illustrating the purification profile of recombinant CIDR1α-PfEMP1 in a variety of imidazole concentrations. The target protein was 27 kDa and resulted in 80 mM imidazole. M: protein marker; E1: soluble fraction in the first elution buffer administration; E2: soluble fraction in the second elution buffer administration.

The recombinant CIDR1α-PfEMP1 was expressed in the soluble fraction, which was purified up to 95% using Ni-NTA agarose. The recombinant protein resolved as a single band of approximately 27 kDa (Figure 1). Calculations using the ProtParam Tool (Swiss Institute of Bioinformatics, Lausanne, Switzerland) showed that the protein consisted of 233 amino acids (the protein started with His-tag and 175 amino acids, and ended with His-tag) [24]. The protein concentration was extrapolated from a Bradford-Assay-based standard protein linear calibration curve using the following equation: y = 0.0078x−0.0703, with an R^2^ value of 0.9962. A total yield of 0.135 mg of recombinant protein/g was obtained from wet *E. coli* cells.

### 3.2. Total Leukocyte and Leukocyte Differential Count

**Figure 2:**
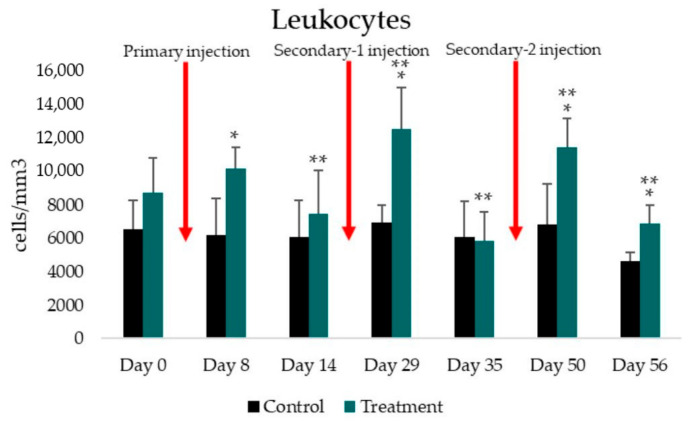
Histogram illustrating the average total leukocyte count in the control and treatment groups. Black box: control group; green box: treatment group; red arrow: time of immunization; *: statistically significant difference between control and treatment groups (*p* < 0.05); **: statistically significant difference between the results of the current treatment and the previous treatment (*p* < 0.05).

The total leukocyte count was measured before immunization and every week after immunization (Day 0, 8, 14, 29, 35, 50, and 56) in the control and treatment groups. The average total leukocyte count is presented in Figure 2. The total leukocyte count increased in the treatment group on Day 8, 29, and 50, whereas it decreased on Day 14, 35, and 56. The highest total leukocyte count was observed on Day 29, as shown in Table 1. The total leukocyte count in the control group was relatively constant, and the lowest total leukocyte count was obtained on Day 56 (4637.50 ± 543.71 cells/mm^3^) (Table 1). The independent *t*-test used to determine the difference between the control and treatment groups indicated a significant difference on Day 8, 29, 50, and 56 (*p* = 0.002, *p* = 0.002, *p* = 0.003, and *p* = 0.004, respectively). The paired *t*-test used to analyze the difference between the results of the current treatment and the previous treatment showed a significant difference in the treatment group on all days except Day 8 (Table 1).

**Figure 3:**
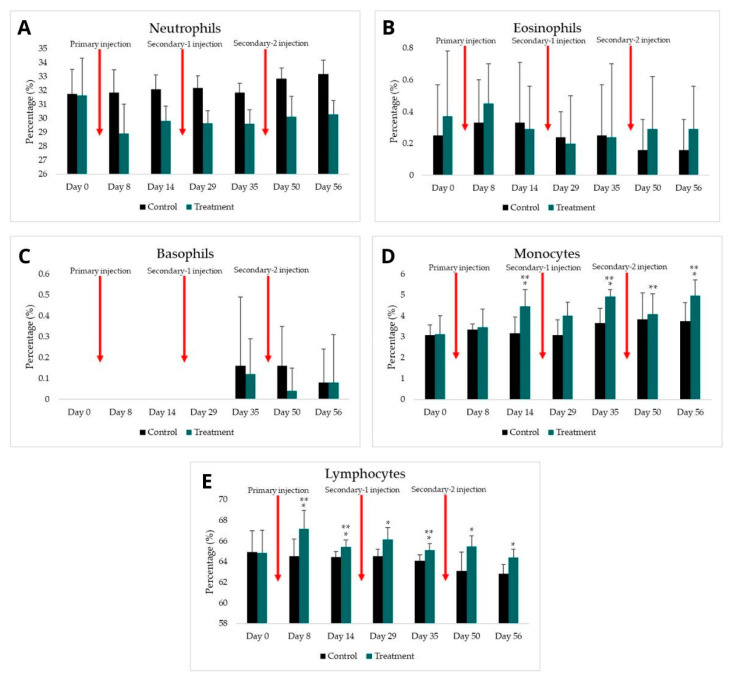
Histogram illustrating the leukocyte differential count percentage. (**A**) Neutrophils; (**B**) Eosinophils; (**C**) Basophils; (**D**) Monocytes; and (**E**) Lymphocytes. Red arrow: time of immunization. *: statistically significant difference between the control and treatment groups (*p* < 0.05); **: statistically significant difference from the previous treatment result (*p* < 0.05).

The leukocyte differential count was performed for five main types of leukocytes, i.e., neutrophils, eosinophils, basophils, monocytes, and lymphocytes, as shown in Figure 3. The mean percentage of neutrophils is presented in Table 1 and Figure 3A. Generally, the percentage of neutrophils in the treatment group was lower than that of the control group. The highest mean neutrophil percentage was observed in the control group on Day 56 (33.16 ± 1.03%), whereas the lowest mean neutrophil percentage was shown in the treatment group on Day 8 (28.91 ± 2.09%). The results of the independent *t*-test, which was used to compare the means of each group, indicated significant differences between the control and treatment groups on all days of measurement (*p* < 0.05). The results of the paired *t*-test, which was performed to compare the current treatment results with the previous treatment results, showed a significant difference in the treatment group only on Day 8 (*p* < 0.017).

The mean percentage of eosinophils and basophils indicated the minimum effect of the recombinant CIDR1α-PfEMP1 protein on the treatment group (Figure 3B,C). Although the percentage of eosinophils increased slightly on Day 8, the percentage of basophils decreased on Day 35, 50, and 56 in the treatment group. Furthermore, the Mann–Whitney test, which was used to compare the difference between the control and treatment groups, and the Wilcoxon test, which was used to compare the current treatment result to the previous treatment result, showed no significant difference on all days of measurement (Table 1).

The differential count for monocytes demonstrated an increase on Day 14, 35, and 56, and a decrease on Day 29 and 50. The highest mean monocyte percentage was found in the treatment group on Day 56 (4.95%), whereas the lowest mean monocyte percentage was found in the control group on Day 0 and 29 (3.08%). The independent *t*-test showed a significant difference between the control and treatment groups on Day 14, 35, and 56 (*p* < 0.05). The paired *t*-test showed a significant difference in the treatment group on Day 14, 35, 50, and 56 (*p* < 0.05).

The mean percentage of lymphocytes is presented in Figure 3E. An increase in the percentage of lymphocytes was found in the treatment group one week after each injection (Day 8, 29, and 50), whereas a decrease was found two weeks after each injection (Day 14, 35, and 56). Overall, the percentage of lymphocytes in the treatment group was higher than in the control group. The highest lymphocyte percentage was shown in the treatment group on Day 8 (67.16%), whereas the lowest lymphocyte percentage was shown on Day 56 (62.83%). The independent *t*-test showed significant differences on all days of measurement (*p* < 0.05). The paired *t*-test showed a significant difference in the treatment group on Days 8, 14, 35, and 56 (*p* < 0.05).

### 3.3. Analysis of IgM Concentration

**Figure 4:**
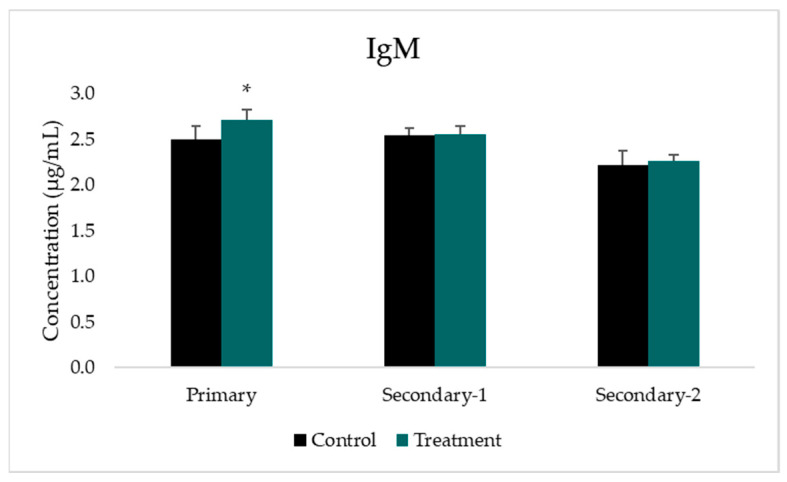
Histogram illustrating the IgM concentration profile. *: statistically significant difference between the control and treatment groups (*p* < 0.05).

The measurements of IgM concentration were conducted three times two weeks after each immunization, i.e., Day 14, 35, and 56, as shown in Table 1 and Figure 4. The concentration of IgM was greatest after the primary immunization (2.72 μg/mL), and decreased after secondary immunization 1 and 2. The Mann–Whitney test showed a significant difference between the control and the treatment group only after primary immunization (*p* < 0.006). There was a slight increase in the concentration of IgM after secondary-1 and secondary-2 immunization in the treatment group. However, this was statistically insignificant when compared to the control group.

## 4. Discussion

The immune response to malaria is complicated. It involves innate and adaptive immunity, which mainly involves leukocytes. This study analyzed total and differential leukocyte counts and IgM concentrations in response to CIDR1α-PfEMP1 recombinant protein immunization. The total leukocyte count in the treatment group was generally higher than the total leukocyte count in the control group (Figure 2). Alassane et al. have also reported an increase in leukocytes in rats infected with *Plasmodium berghei* [25]. This increase in leukocytes indicates an immune response to the CIDR1α-PfEMP1 protein. The protein stimulates the production of proinflammatory cytokines, leading to an inflammatory reaction and leukocytosis [26].

The differential leukocyte counts indicated that specific leukocyte cells respond differently to the CIDR1α-PfEMP1 protein. The percentage of neutrophils, eosinophils, and basophils tended to decrease in the treatment group, but the percentage of monocytes and lymphocytes increased. Darlina et al. [27] and Oyewusi et al. [28] reported a decrease in the percentage of neutrophils in mice infected with *P. berghei*. This low neutrophil percentage indicates the use of this cell in an immune response against a foreign particle, i.e., the *Plasmodium* antigen. Neutrophils, as the first line of defense, can express intercellular adhesion molecules (ICAMs) and EPCR receptors on their surface so that these receptors can bind to the PfEMP1 domain. This process mediates the attachment and ingestion of foreign particles [29]. Phagocytosis induces neutrophil apoptosis. This is known as phagocytosis-induced cell death (PICD). During phagocytosis, the neutrophils will produce NADPH oxidase-derived reactive oxygen species (ROS), proteases, and antimicrobial peptides, which create a lethal intraphagosomal environment, resulting in a low percentage of neutrophils [30].

The percentage of eosinophils and basophils in the treatment group was not significantly different from the control group. This result is supported by previous studies conducted by Sumah et al. [31] and Cahyaningsih et al. [32], who reported that a *P. berghei* infection did not affect the percentage of eosinophils and basophils in mice. Previous studies have demonstrated the indirect role of eosinophils and basophils in malaria immunity. The active role of eosinophils in malaria infection is initiated by a protein that is secreted by *P. falciparum*, i.e., *P. falciparum* translationally controlled tumor protein (PfTCTP), which is a histamine-releasing factor (HRF) homolog. PfTCTP is able to induce the release of histamines, chemotaxis, and IL-8 by eosinophils [33]. This study indicated that the CIDR1α-PfEMP1 protein does not initiate eosinophil activity, as well as PfTCTP. Similar to eosinophils, basophils play a minimal role in malaria immunity. The activation of basophils is caused by specific conditions, such as nematode infections and hypersensitivity, and is not associated with protozoal infections [34].

The mean percentage of monocytes in the treatment group increased, especially two weeks after immunization, and increased along with the injection frequency (Table 1). The increased observed in the percentage of monocytes 14 days after each immunization is in accordance with the role that monocytes play in the second line of defense and with the reduction in neutrophils observed 8 days after each immunization [35]. These findings are similar to previous studies that evaluated the immune response in mice infected with *P. berghei* [31,32]. The activation of transcription factors, cytokines, and chemokine responses in monocytes can be stimulated by immunomodulatory molecules, such as PfEMP1 [36]. An increase in the number of monocytes is related to their capacity to develop a memory for malaria infection in order to prepare its metabolism, chromatin, and Toll-like receptor (TLR) expression in order to recognize the parasites if malaria re-infection occurs [11].

The lymphocyte percentage increased and decreased over time due to CIDR1α-PfEMP1 protein immunization. Significant increases in the number of lymphocytes occurred after a week of injection, but decreased two weeks after protein injection. Lymphocytes are activated when antigens are presented to CD4+ T-cells via MHC-II molecules [37]. These cells initiate B-cell maturation and CD8+ T-cell activation in order to produce antibodies and cytokines to eliminate the parasites [13]. Therefore, the number of circulatory lymphocytes will increase in the early phase of antigen exposure. A decrease in lymphocytes is caused by the migration of lymphocytes from the blood circulation to the tissues [38]. Mature lymphocytes in the primary lymphoid organs subsequently enter the blood circulation and the secondary lymphoid organs. In the secondary lymphoid organs, an adaptive immune response occurs, which involves binding between antigens and lymphocytes via APCs, such as macrophages and dendritic cells [39].

The IgM concentration was measured two weeks after each CIDR1α-PfEMP1 protein injection, and the highest IgM concentration was observed after the primary immunization. A previous study reported that the CIDR1α domain induces B-cell activating factor (BAFF), resulting in the activation and proliferation of B-cells and antibody secretion [40]. However, several factors affect the immunogenicity of CIDR1α-PfEMP1, including its molecular weight and dose. A protein with a high molecular weight (greater than 100 kDa) is a potent immunogen [39]. The minimal dose of a purified antigen needed to induce an immune response in rats is 100 μg, but each antigen has a requisite dose range in order to induce the optimal immune response [37,41]. This study was performed using a dose of 150 μg/μL. A low CIDR1α-PfEMP1 molecular weight of approximately 27 kDa could reduce its immunogenicity. However, the use of an adjuvant potentially overcomes this limitation.

## 5. Conclusions

The CIDR1α-PfEMP1 recombinant protein induces the innate immune response, which is characterized by an increase in the total number of leukocytes, the percentage of monocytes, and the percentage of lymphocytes, and a decrease in the percentage of neutrophils. It also induces the adaptive immune response, which is characterized by an increase in IgM concentration. Therefore, CIDR1α-PfEMP1 is a potential malaria vaccine candidate.

## Figures and Tables

**Table 1:** The total number of leukocytes, the leukocyte differential count, the IgM concentration, and the statistical analysis.

Indicator		Pre-Injection Day 0	Primary Injection		Secondary-1 Injection		Secondary-2 Injection	
			Day 8	Day 14	Day 29	Day 35	Day 50	Day 56
Total leukocyte number								
Control (cells/mm^3^)		6562.52 ± 1752.31	6187.50 ± 2205.06	6112.50 ± 2177.29	6937.50 ± 1068.00	6087.50 ± 2125.78	6837.50 ± 2463.18	4637.50 ± 543.71
Treatment (cells/mm^3^)		8756.25 ± 2066.30	10187.50 ± 1282.50	7481.25 ± 2598.89	12581.25 ± 2490.25	5881.25 ± 1739.84	11462.50 ± 1743.71	6900.00 ± 1115.15
*p*-value	to control group	0.100	0.002 *	0.389	0.002 *	0.860	0.003 *	0.004
	to previous treatment	-	0.113	0.012 **	0.002 **	0.001 **	0.001 **	0.001 **
Neutrophils								
Control (%)		31.75 ± 1.77	31.83 ± 1.66	32.08 ± 1.03	32.16 ± 0.88	31.83 ± 0.69	32.83 ± 0.79	33.16 ± 1.03
Treatment (%)		31.66 ± 2.64	28.91 ± 2.09	29.83 ± 1.05	29.66 ± 0.87	29.62 ± 0.99	30.12 ± 1.45	30.29 ± 0.98
*p*-value	to control group	0.954	0.036 *	0.006 *	0.001 *	0.003 *	0.007 *	0.001 *
	to previous treatment	-	0.017 **	0.154	0.615	0.925	0.304	0.810
Eosinophils								
Control (%)		0.25 ± 0.32	0.33 ± 0.27	0.33 ± 0.38	0.24 ± 0.16	0.25 ± 0.32	0.16 ± 0.19	0.16 ± 0.19
Treatment (%)		0.37 ± 0.41	0.45 ± 0.25	0.29 ± 0.27	0.20 ± 0.30	0.24 ± 0.46	0.29 ± 0.33	0.29 ± 0.27
*p*-value	to control group	0.644	0.409	0.856	0.578	0.702	0.580	0.463
	to previous treatment	-	0.680	0.245	0.157	1.000	0.498	0.798
Basophils								
Control (%)		0.00 ± 0.00	0.00 ± 0.00	0.00 ± 0.00	0.00 ± 0.00	0.16 ± 0.33	0.16 ± 0.19	0.08 ± 0.16
Treatment (%)		0.00 ± 0.00	0.00 ± 0.00	0.00 ± 0.00	0.00 ± 0.00	0.12 ± 0.17	0.04 ± 0.11	0.08 ± 0.23
*p*-value	to control group	1.000	1.000	1.000	1.000	0.919	0.176	0.695
	to previous treatment	-	1.000	1.000	1.000	0.083	0.317	0.317
Monocytes								
Control (%)		3.08 ± 0.49	3.33 ± 0.27	3.16 ± 0.79	3.08 ± 0.73	3.66 ± 0.71	3.83 ± 1.26	3.75 ± 0.87
Treatment (%)		3.12 ± 0.89	3.45 ± 0.87	4.45 ± 0.81	4.00 ± 0.66	4.91 ± 0.34	4.08 ± 0.98	4.95 ± 0.76
*p*-value	to control group	0.936	0.790	0.026 *	0.055	0.002 *	0.714	0.033 *
	to previous treatment	-	0.534	0.012 **	0.329	0.22 **	0.036 **	0.039 **
Lymphocytes								
Control (%)		64.91 ± 2.07	64.50 ± 1.69	64.41 ± 0.56	64.50 ± 0.69	64.08 ± 0.57	63.00 ± 1.86	62.83 ± 0.88
Treatment (%)		64.83 ± 2.19	67.16 ± 1.77	65.41 ± 0.68	66.12 ± 1.18	65.08 ± 0.63	65.45 ± 1.03	64.37 ± 0.80
*p*-value	to control group	0.950	0.032 *	0.031 *	0.031 *	0.024 *	0.011 *	0.013 *
	to previous treatment	-	0.031 **	0.019 **	0.131	0.006 **	0.369	0.017 **
IgM concentration								
Control (μg/mL)		-	-	2.50 ± 0.15	-	2.54 ± 0.08	-	2.22 ± 0.15
Treatment (μg/mL)		-	-	2.72 ± 0.12	-	2.56 ± 0.08	-	2.26 ± 0.07
*p*-value to control group				0.006 *		0.732		0.607

* statistically significant difference between control and treatment groups. ** statistically significant difference from the previous treatment result.

## Data Availability

Not applicable.
